# Conserved Regulatory Pathways for Stock-Scion Healing Revealed by Comparative Analysis of Arabidopsis and Tomato Grafting Transcriptomes

**DOI:** 10.3389/fpls.2021.810465

**Published:** 2022-02-24

**Authors:** Lulu Xie, Jianfan Tian, Lixin Peng, Qingqing Cui, Yang Liu, Jiyang Liu, Fu Li, Siyuan Zhang, Jianchang Gao

**Affiliations:** ^1^Key Laboratory of Horticultural Crop Biology and Germplasm Innovation (Ministry of Agriculture), Institute of Vegetables and Flowers, Chinese Academy of Agricultural Sciences, Beijing, China; ^2^Shouguang Vegetables Research and Development Center, Chinese Academy of Agricultural Sciences, Shouguang, China; ^3^Institute of Modern Agricultural Research, Dalian University, Dalian, China

**Keywords:** graft healing, stock-scion healing, tomato, Arabidopsis, comparative transcriptomics, evolutionarily conserved mechanisms

## Abstract

Many plants can successfully join root and shoot sections at cut surfaces when severed at the stem. Graft healing is complex and conserved in diverse taxonomic groups with different vascular structures. Herein, we compared transcriptome data from autografted and separated stem sections of *Arabidopsis thaliana* and tomato (*Solanum lycopersicum*) to explore changes related to graft healing. Using orthologous gene pairs identified between the two species, temperal expression patterns of evolutionary associated genes in grafted top and bottom, separated top and bottom, and intact stems were exhibited. Genes with expression preference indicate functional diversification of genes related to anatomical structure and cellular development in the two species. Expression profiles of the variable genes revealed common pathways operating during graft healing, including phenylpropanoid metabolism, response to oxygen-containing compounds, xylan, and cell wall biogenesis, mitosis and the cell cycle, carboxylic acid catabolism, and meristem structural organization. In addition, vascular differentiation related NAC domain transcription factors and genome-wide members in Arabidopsis and tomato were used for phylogenetic and expression analysis. Expression differences were largely consistent with sequence differences, reflecting high similarity for protein-coding and regulatory regions of individual clades. NAC proteins mainly clustered in accordance with their reported functions in xylem differentiation or cambium formation. The putative conserved mechanisms suggested by conserved genes and functions could help to expand graft healing theory to a wider range of species, and temporal fluctuations in common pathways imply conserved biological processes during graft healing.

## Introduction

Many plants possess the ability to reunite separated root and shoot parts at the cut surface when severed at the stem. The above healing ability was employed *via* the ancients to create grafted chimeric plants, which combine the ideal characteristics of stock and scion (Goldschmidt, 2014; Wang et al., 2017). The grafting technique is now widely applied to improve the disease resistance and yield of vegetable crops together with tree species.

The graft healing process involves complicated and interlinked physiological events, including wounding stress responses, callus formation, cell communication between scion and stock, and regeneration and reunion of vascular bundles. Much effort has been expended at different levels to better understand the mechanisms that underlie graft healing. Cytologically, some fine changes occur at the junction surface; parenchyma cells proliferate and fill the gap between scion and stock, plasmodesmata interconnect, and vascular differentiation takes place, as demonstrated by early studies on *Pisum sativum*, *Solanum pennellii*, and *Vicia/Helianthus* (Stoddard and McCully, 1979; Moore, 1984; Kollmann and Glockmann, 1985). Physiologically, phytohormones and phytohormone-dependent and -independent gene regulatory networks are recruited in each of the physiological events. Plant hormones associated with stress for instance ethylene and jasmonic acid are activated within hours of injury, which triggers callus generation and defense response (Yin et al., 2012; Huang et al., 2017). The cytokinin production and signal transduction facilitate callus generation through regulating cell cycle proteins (Ikeuchi et al., 2017). The most important signal response pathways, sugars and auxin, play pivotal roles in the communication of cell between stock and scion, and in specifying vascular patterns (Melnyk et al., 2015, 2018). In addition, graft incompatibility is usually accompanied by the accumulation of phenylalanine pathway metabolites and abnormal cell wall modification (Pina and Errea, 2008; Loupit and Cookson, 2020). Only changes in the functions of β-1,4-glucanases, which facilitate cell wall reconstruction near the graft interface, strengthen graft affinity for a diverse range of angiosperms (Notaguchi et al., 2020).

Transcriptome sequencing is employed as an useful tool to explore the graft healing genetics. RNA sequencing (RNA-seq) transcriptome together with microarray analysis of Arabidopsis and the woody plants for instance pecan, hickory, and grape exhibited that genes were participated in jasmonic acid and ethylene biosynthesis, reactive oxygen species scavenging, auxin transport and signaling, the elongation and proliferation of cell, cell wall modification, and vascular differentiation undergo significant differential expression during graft union development (Yin et al., 2012; Cookson et al., 2013; Qiu et al., 2016; Mo et al., 2018). Furthermore, research on Arabidopsis has unveiled detailed spatial and temporal dynamic (Melnyk et al., 2018). In contrast to non-grafted samples, genes with similar high level expression in the grafted samples were principally enriched in defense and stress response pathways in the early stage. About 5 days after transplantation, pathways associated with vascular differentiation and cell wall tissue were increased in transplantation samples (containing top and bottom samples).

Some members of the NAC domain-containing superfamily play graft healing-related roles, and are often used as markers of vascular differentiation. NAC genes are plant-specific, named from NAM, ATAF, and CUC, and they manipulate developmental and stress-induced responses (Aida et al., 1997; Ooka et al., 2003). As revealed by vascular differentiation patterns of primary root and leaf veins in Arabidopsis, NAC101/VND6 and NAC030/VND7 are key regulators of xylem differentiation (Kubo et al., 2005), whereas NAC020, NAC045, and NAC086 participate in phloem differentiation (Furuta et al., 2014; Kondo et al., 2016). In addition, NAC071 and NAC096 transmit auxin and ethylene signals, triggering xyloglucan endotransglucosylase/hydrolase cell wall organization, and promoting cambial cell formation after wounding (Pitaksaringkarn et al., 2014; Matsuoka et al., 2021). Although these patterns were observed in Arabidopsis, there are similar patterns in other species (Melnyk et al., 2018; Cui et al., 2021).

The graft healing process is conserved in diverse taxonomic groups with different vascular structures. This may reflect inheritance from the common ancestor of vascular plants, and persists in most extant plants. Thus, we can use these common mechanisms in grafting applications, as well as solanaceous and cucurbitaceous vegetable production. Although grafting is widely applied in tomato (*Solanum lycopersicum*) production, the molecular mechanism of graft union formation in tomato plants remains obscure. Mechanisms related to the graft healing process have been investigated in Arabidopsis as a model plant. Characterization of conserved genes and functions in different plants could help to decipher pathways and reveal to what extent they can be applied to tomato and other plants. In this study, we collected transcriptome data at identical timepoints from autografted and separated stem sections of *Arabidopsis thaliana* and tomato to perform a comparative analysis of transcriptomic changes related to graft healing.

## Materials and Methods

### Transcriptome Data and Analysis

**FIGURE 1:**
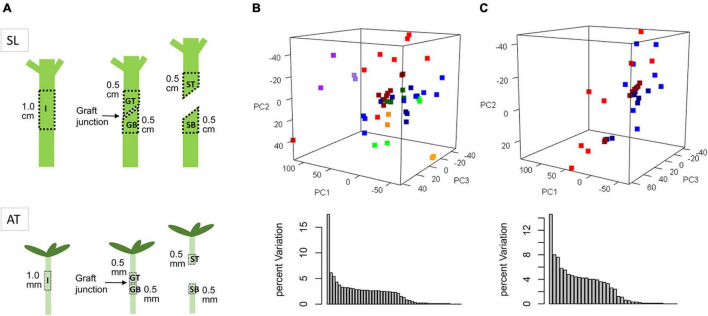
Diagram of sampling regions **(A)** and principle component analysis (PCA) of transcriptomic variation of all samples **(B)** or GT and GB samples only **(C)**. SL and AT are short for *Solanum lycopesicum* and *Arabidopsis thaliana*, respectively. I, GT, GB, ST, and SB represent sample types of intact, grafted top, grafted bottom, separated top, and separated bottom. Red dots, GT_AT; blue, GB_AT; purple, ST_AT; orange, SB_AT; green, I_AT; dark red, GT_SL; dark blue, GB_SL; dark purple, ST_SL; dark orange, SB_SL; dark green, I_SL.

The grafted plants we used were Arabidopsis scion grafted on Arabidopsis rootstock and tomato scion grafted on tomato rootstock (Figure 1A). Grafted and intact three-leaf-stage seedlings of tomato “Jiulv 787” were used for sampling at different timepoints. A 5 mm length of stem was taken above or below the graft junction from grafted plants, and 10 mm sections were taken from intact plants. We used intact stem sections of non-grafted plants as control. Those control plants were grown at the same circumstance as grafted plants, and were sampled at four time points evenly from start to finish of the experiment. For each sample, 15–20 stem sections were pooled for library preparation and RNA extraction through employing the NEBNext Ultra RNA Library Prep Kit (New England Biolabs, Ipswich, MA United States). HiSeq 2000 platform or Illumina NovaSeq 6000 (Novogene Bio Tech Co., Ltd.) was utilized to acquire the paired-end reads. The detailed information of Arabidopsis grafting can be found in the reference (Melnyk et al., 2018).

From TAIR^1^ and Phytozome^2^ databases, we downloaded the annotation files and reference genome sequences of *A. thaliana* (v10) and *S. lycopersicum* (v2.4), respectively. With the aim of processing original sequencing reads, NGSQCToolkit v2.3.3 (Patel and Jain, 2012) was employed to delete reads with many low-quality bases (with a Phred quality score less than 20) more than 30% (−l 70, −s 20) or the paired-end reads involving ambiguous bases (Ns). From the filtered reads, the first 10 unstable bases were cut, and the clean reads were subsequently mapped to reference genome sequence of *S. lycopersicum* through employing Hisat2 in default settings (Kim et al., 2015). After duplications were deleted through SAMtools v0.1.19 (Li et al., 2009), in accordance with general feature format files, residual reads were assembled. In each sample, the relative abundance of each transcript (fragments per kilobase of exon per million fragments mapped; FPKM) was normalized and estimated *via* Cuffnorm and Cuffquant in a software package of Cufflinks (v2.2.1) (Trapnell et al., 2010). The values of FPKM were scaled by the median of the fragment counts geometric means in all of the libraries.

Raw data of RNA-seq transcriptomes of tomato and Arabidopsis can be obtained by the database of NCBI Sequence Read Archive (SRA) with the BioProject numbers PRJNA419306, PRJNA528328, and PRJNA645644.

### Identification of Orthologous Gene Pairs

The reciprocal best hit (RBH) method was used to identify orthologous gene pairs. Sequences of *S. lycopersicum* were first used as queries for BLAST searches against the *A. thaliana* database, and queries and databases were then exchanged. In both cases, tBLASTx tool (Altschul et al., 1990) was employed with a threshold of 1e-5. Where the top hits of the two BLAST processes were identical, the two genes were regarded as an orthologous gene pair. 11,787 orthologous gene pairs of Arabidopsis and tomato genes were obtained (Supplementary Table 1).

### Principle Component Analysis and Visualization

After *z*-score transformation, PCA of samples was performed by the prcomp() function in R (R Core Team, 2018). A bar plot of the principle component percentage distribution was drawn using the ggplot2 package (Wickham, 2009). Using the first three principle components, three-dimensional visualization was conducted using the rgl package (Murdoch and Adler, 2021).

### Gene Co-expression Network Analysis

Using all orthologous gene pairs, each contains one Arabidopsis gene and one tomato gene, the top 5,000 variable orthologous gene pairs were obtained using the FindVariableGenes function and the *vst* method in the R package SEURAT (Satija et al., 2015; Supplementary Figure 1A). In the matrix of expression levels, the rows are orthologous gene pairs, and the columns are different sample types of Arabidopsis or tomato. Log2-transformed FPKM values were analyzed *via* applying R package WGCNA (Langfelder and Horvath, 2008) for gene co-expression networks. First of all, through employing the approximate scale-free topology criterion, the PickSoftThreshold function was applied for selecting a value of soft threshold (power). We generate the scale-free topological fitting indices for distinct powers. An appropriate power was selected, the signed R^2^ threshold is greater than 0.8 (Supplementary Figure 1B). A weighted co-expression clusters (modules) was subsequently acquired *via* utilizing automatic network building function blockwiseModules, with the below calculation settings: power = 9, networkType = signed, corType = bicor, minModule-Size = 100, mergeCutHeight = 0.15).

### Gene Annotation and Enrichment Analysis

AmiGO (The Gene Ontology Consortium, 2000) and ClueGO (Bindea et al., 2009) was respectively applied for the Gene Ontology (GO) annotation together with overrepresentation analysis of biological processes. AmiGO annotation was conducted *via* utilizing genome-stable IDs of Arabidopsis as the queries. *p*-values were acquired by Fisher’s exact detections *via* using AmiGO, which was then corrected through calculating false discovery rate (FDR). ClueGO was also used for GO annotation. ClueGO nets were set as “medium” and their connectivity was on the basis of 0.4 Kappa score. Bonferroni step-down strategy together with Bilateral hypergeometric test were employed for the correction of *p*-value. The GO item grouping parameter sets the initial group size to 1, group merge to 50%, and the leading group item according to the highest significance.

### Identification of NAC Transcription Factor Homologs

Protein sequences in Arabidopsis (v10) and tomato (v2.4) genomes were downloaded from TAIR and Phytozome, respectively. BLASTP (Altschul et al., 1990) and HMMER (Eddy, 2009) were applied for determining the NAC domain proteins according to homology searches. For BLAST searches, protein sequences from the literature (Ooka et al., 2003; Kondo et al., 2016) were used as queries, and the acquiring hits were filtered through *E*-value of 1e-5. For the searches of HMMER, the target protein database was searched *via* using the.hmm file (PF02365) acquired from PFAM database^3^ as a query. With the threshold *E*-value of 0.01. Sequences containing complete NAC domains were extracted (Supplementary Table 2).

### Phylogenetic Tree Construction and Expression Pattern Visualization

Protein sequences were aligned with MAFFT (Katoh et al., 2002), followed by manual adjustment. Only conserved NAC domain sequences were used for phylogenetic tree construction. MEGA (Tamura et al., 2011) chose the most suitable amino acid substitution model JTT + G + I. The analyses of maximum likelihood (ML) were conducted by RAxML (Stamatakis et al., 2008) with 1,000 bootstrap replicates. Expression pattern visualization of the tree data was performed using iTol (Letunic and Bork, 2016).

### Statistical Analysis

Kappa consistency test was used to determine the inter-species consistency of expression levels among different clustering clade, or different sample types within the same clade. Using the rank order of expression levels, weighted Kappa values and consistency test was performed by R package vcd (Meyer et al., 2021), with weights set as equal-spacing.

## Results

### Transcriptomic Variation of Orthologous Pairs Between Arabidopsis and Tomato

According to our previous work as well as the Arabidopsis grafting literature, it can be confirmed that the vascular reconnection accomplished within 10 days after grafting in both Arabidopsis and tomato (Melnyk et al., 2018; Cui et al., 2021). And the cytologically changes at the junction surface, including parenchyma cells proliferate, vascular differentiation, and vascular reconnection, are similar and synchronous. Based on the phenotypic changes, transcriptome data for grafted samples were collected from corresponding tissue types and timepoints for both species, including grafted top and grafted bottom samples at 6, 12, 24, 48, 72, 120, 168, and 240 h after grafting (HAG; named GT006, GB006, GT012, GB012, etc.), separated top and separated bottom samples at 48 and 72 HAG (named ST048, SB048, ST072, and SB072), and intact samples (I000, I072, etc.), to perform inter-species comparison at the transcriptomic level (Figure 1A and Supplementary Table 1).

Following reciprocal best hit BLAST searches, 11,787 orthologous pairs of Arabidopsis and tomato genes were obtained and used for further analysis (Supplementary Table 1). Among them, genes of 312 orthologous pairs were not expressed in any samples from either species. Functional annotation of the 320 orthologous pairs that were only expressed in Arabidopsis showed that the enriched functions were anatomical structure development (GO:0048856) and cellular developmental process (GO:0048869). Meanwhile, functional annotation of the 498 orthologous pairs that were only expressed in tomato showed that the enriched functions were anatomical structure development (GO:0048856) and multicellular organism development (GO:0007275). Interestingly, both groups include anatomical structure and cellular development processes, suggesting functional diversification of these genes in the two species.

Differences in genetic background, tissue anatomy, environment, and sequencing procedures can result in differences in average expression values of genes in many ortholog pairs between two species. Therefore, *z*-score transformation can be performed on samples of the same species, and the resulting data can be subjected to principle component analysis (PCA). The three-dimensional view of PC1 (17.5%), PC2 (6.1%) and PC3 (5.4%) showed that samples of different tissue types were dispersed, whereas samples of the same tissue types and species tended to be clustered (Figure 1B). This suggests that genetic background and sample type were the two main factors causing large variation. However, this is likely to mask variation in the graft healing process. Therefore, only grafted samples (GT and GB) were used for further analysis, and scaled again within the same species, resulting in a relatively gradual transition in the sample distribution (Figure 1C).

### Common Pathways in Arabidopsis and Tomato During Graft Healing

**FIGURE 2:**
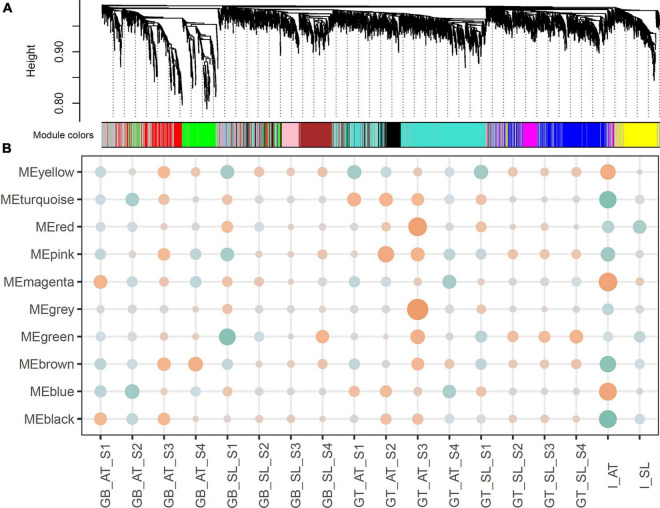
**(A)** Hierarchical clustering and co-expression modules. The hierarchical cluster tree shows gene co-expression modules identified by WGCNA. The branches constitute 10 modules labeled in different colors. **(B)** Relationships between modules and traits. Correlation coefficients are shown by color (orange for positive and green for negative). The size represents the degree of significance, calculated as −log2 (*p*-value).

Using the grafted top and grafted bottom series data, the weighted gene co-expression analysis was implemented to identify pathways common to both species during graft healing. Expression profiles of 5,000 variable genes were classified as ten modules (MEs), exhibiting 9 various co-expression networks, namely MEturquoise (1,023), MEblue (683), MEbrown (428), MEyellow (386), MEgreen (368), MEred (320), MEblack (276), MEpink (258), MEmagenta (190), and MEgray (1,068) for outliers that do not belong to any cluster (Figure 2A).

We then associated co-expression modules with certain traits. As reported in previous studies, the process of graft healing exhibited stage-specific features (Cui et al., 2021). According to the regular clustering pattern of samples based on the sample type and the time after grafting, four stages were set up as developmental traits: S1 for 6 and 12 HAG, S2 for 24 and 48 HAG, S3 for 72 and 120 HAG, and S4 for 168 and 240 HAG. In the association analysis, the value of each concerned trait was set to 1, and others were set to 0. Due to the assigned network type, the directions of correlation between modules (the rows) and traits (the columns) can reflect expression features of orthologous genes in each module (Figure 2B). For example, MEbrown was negatively correlated with the trait of GB_AT_S1 and S2, then became positive correlation at S3 and S4. The similar situation also exhibited in GB_SL samples. It suggested the ascending change of these MEbrown genes in both GB_AT and GB_SL samples (Figure 2B and Supplementary Figure 2). Some modules exhibited very similar patterns in the two species, such as MEbrown, MEgreen, MEblack, MEpink, and MEmagenta. Meanwhile, other modules showed diverse expression patterns in the two species. Combined with GO annotation of each module (Table 1), this allowed common pathways active in Arabidopsis and tomato during graft healing to be predicted. Genes in MEbrown were expressed highly at S3 and S4 in both GT and GB samples of both species, and their enriched functions were phenylpropanoid metabolic process (GO: 0009698) and response to oxygen-containing compounds (GO: 1901700). For MEgreen, xylan and cell wall biogenesis were the main enriched functions, and they were first expressed at S2 in GT samples and at S3 in GB samples. MEblack and MEpink both had expression peaks at S2 and S3 stages, especially in GT samples, and they participate in mitosis and the cell cycle (GO: 0007049). The enriched functions of MEmagenta were carboxylic acid catabolic process (GO: 0046395) and meristem structural organization (GO: 0009933), reflecting changes at the top or bottom positions after grafting to intact tissue. The results suggest that these common pathways may be active in Arabidopsis and tomato during graft healing. Meanwhile, other modules including MEturquoise, MEblue, and MEyellow were mainly associated with primary metabolism. Additionally, expression behavior and species preferences were more pronounced for AT and SL. These pathways may also play an indispensable role in graft healing, but reflect specialization in different taxa.

**TABLE 1:** Gene Ontology (GO) enrichment of each module.

Module	GO term	GO number	*P*-value
MEturquoise			
	Cellular component organization or biogenesis	GO:0071840	3.55E-12
	Response to organic substance	GO:0010033	4.41E-05
MEblue			
	Macromolecular complex subunit organization	GO:0043933	3.10E-05
	Protein localization	GO:0008104	9.60E-04
**MEbrown**			
	Phenylpropanoid metabolic process	GO:0009698	3.63E-05
	Response to oxygen-containing compound	GO:1901700	6.50E-05
MEyellow			
	Response to light stimulus	GO:0009416	5.51E-15
	Photosynthesis, light reaction	GO:0019684	8.02E-15
**MEgreen**			
	Xylan metabolic process	GO:0045491	1.00E-02
	Plant-type secondary cell wall biogenesis	GO:0009834	1.22E-02
MEred			
	None		
**MEblack**			
	Mitotic spindle assembly checkpoint	GO:0007094	8.00E-05
	Histone modification	GO:0016570	1.60E-03
**MEpink**			
	Microtubule-based movement	GO:0007018	6.43E-13
	Cell cycle	GO:0007049	3.78E-09
**MEmagenta**			
	Meristem structural organization	GO:0009933	4.20E-03
	Carboxylic acid catabolic process	GO:0046395	4.70E-03

*Modules with common pathways were presented in bold font. The two most significant terms were displayed.*

### Temporal Variation Characteristics of Common Pathways in Arabidopsis and Tomato During Graft Healing

**FIGURE 3:**
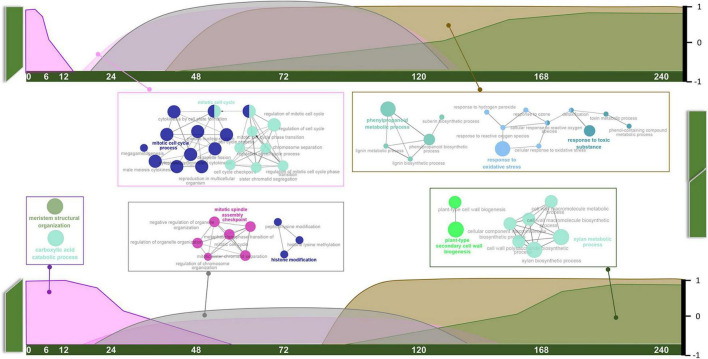
Schematic diagram of temporal variation characteristics and GO annotations of common pathways.

As mentioned above, orthologous pairs in each module displayed specific patterns regarding tissue type and developmental stage. Based on the expression patterns of every module with detailed information (Supplementary Figure 2), combined with GO annotation, we arranged them on a time scale and present the results in the form of a schematic diagram (Figure 3).

Common pathways appear to play roles in three stages. Carboxylic acid catabolic process and meristem structural organization (MEmagenta) mainly functioned at the S1 stage. These genes were highly expressed in intact tissue, suggesting roles in stem development. Their expression levels gradually declined along with the healing process. Mitosis and histone modification pathways (MEblack) and cell cycle-related pathways (MEpink) overlapped at S2 and S3 stages, and the genes expression levels were much higher in GT samples than in GB samples. Phenylpropanoid metabolic and oxygen-containing compound response pathways (MEbrown) and xylan and cell wall biogenesis pathways (MEgreen) were prevalent at S3 and S4 stages, and also overlapped. Interestingly, expression levels of these pathway genes always increased in GT samples before GB samples.

### Vascular Differentiation Patterns Reflected by Phylogeny and Expression Analysis of NAC Family Proteins

**FIGURE 4:**
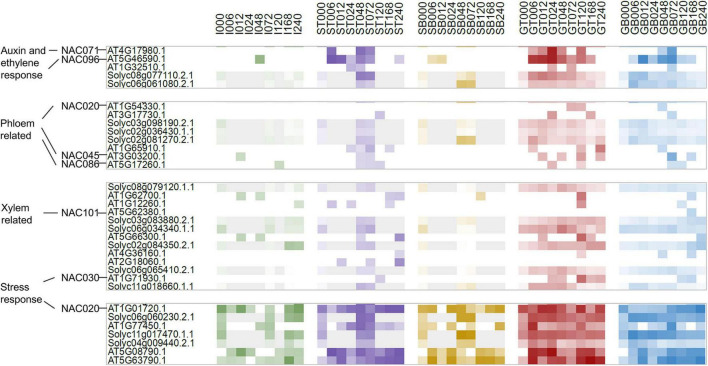
Cluster relationship and expression patterns of NAC homologs.

For graft healing to be considered a success, vascular tissue differentiation and reconnection must occur during the sampling period. We therefore observed the genome-wide phylogeny and expression features of NAC family proteins, since some members are often used as markers for xylem, phloem, and cambium formation. Conserved NAC domain sequences of 109 Arabidopsis and 88 tomato proteins were used for phylogenetic tree construction. They clustered into 8 clades, named c1—c8. The transcriptomic abundances for all samples are presented along the tree (Supplementary Figure 3). It appears that there is a great consistency of the differences between expression levels and sequence similarity, even if the data are of various types and gathered from distinct species. By testing the consistency of average expression levels of eight clustering clades between two species, the Kappa value is 0.8095 (*P* = 1.782e-10). Furthermore, expression levels of different sample types in clades c2, c3, c4, and c7 also exhibit significant consistency (Supplementary Figure 4). This reflects the high similarity of both protein-coding and regulatory regions of individual clades. Except for pseudogenic clades with extremely low abundance and housekeeping clades with constant expression, NAC proteins clustered in accordance with their reported functions (Figure 4). NAC101/VND6 and NAC030/VND7 are clustered together in one, and were expressed in GT, GB, and ST samples. NAC020 and NAC086 were found to be expressed successively when phloem develops in primary roots and leaf veins (Furuta et al., 2014; Kondo et al., 2016), but there was no strong consistency in any sample types over time. While in the clade adjacent to NAC020, auxin and ethylene response genes NAC071 and NAC096 displayed obvious pattern based on sample type and time. In addition, NAC002/ATAF1-containing clade genes were expressed highly in all samples, but there was no apparent correlation with the advancement of graft healing, implying their functions only in stress responses independent of graft healing.

## Discussion

### Putative Conserved Mechanisms Reflected by Orthologous Gene Pairs Expand Graft Healing Theory to a Wider Range of Species

Orthologs are the genes evolved from a common ancestor gene in various species. They usually maintain the same function in the process of evolution. When comparing a pair of orthologs, the function may be conserved, obtained, or lost. Some conserved regulatory mechanisms, such as the ABC model of flower development and the flavonoid pathway MBW ternary complex, have brought convenience for research and been applied widely in angiosperms (Coen and Meyerowitz, 1991; Xu et al., 2015).

Regarding graft healing, many previous studies revealed recurring patterns in different species, suggesting conserved mechanisms. To explore the putative conserved mechanisms of graft healing, we compared orthologs in the present work. The results revealed similar top hits for gene pairs, confirming conserved gene structures. Secondly, similar expression patterns in both species reflected conserved regulatory pathways responsible for these phenotypic changes. We identified common pathways, in terms of both structure and expression, including phenylpropanoid metabolic process, oxygen-containing compounds, xylan and cell wall biogenesis, mitosis and cell cycle, and carboxylic acid catabolic process, all of which are worthy of further investigation. Additionally, orthologs could be used in other systems.

However, there are likely to be other conserved pathways that we did not identify. Data handling of normalization tends to reveal long-term changes while missing short-term differences. Several studies have reported that the wounding response and auxin signaling occur in the early stages after grafting (Yin et al., 2012; Melnyk et al., 2015; Xie et al., 2019). Both are inevitable processes after cutting. In particular, the auxin gradient provides positional information, and this mode of action may vary in different systems.

### Temporal Fluctuations in Common Pathways Suggest Conserved Biological Processes During Graft Healing

Graft healing begins immediately after cutting of intact stems, and terminates at the recovery of normal transport function. During this period, large changes occur near the graft surface of both the stock and the scion. Our results suggest that the overall process is composed of three phases. The first phase (phase I, < 48 HAG) occurs in intact tissue, and declines gradually after grafting (Figure 3). The rate of decline in GB tissues is slower than in GT tissues. Genes in this module were enriched in pathways related to meristem structural organization and carboxylic acid catabolic process. The former is believed to function in normal growing stems, and the latter supplies energy for development. Lagging ∼24 h behind phase I in both GB and GT samples, phase III (> 72 HAG) begins and increases continuously until healing is complete. This phase involves genes related to the phenylpropanoid metabolic process, response to oxidative stress, plant-type secondary cell wall biogenesis, and xylan metabolism, all of them are related to secondary metabolic pathways or development. Between these two phases, phase II (24–120 HAG) occurs at the same time in GT and GB. The main events in this phase are mitosis and epigenetic changes such as histone modification. Transitions between the three phases, characterized by common pathways, imply conserved regulatory mechanisms in different species.

Vascular differentiation is a hallmark event of graft healing, but no related pathways were identified by co-expression analysis of orthologous pairs. As an alternative, we explored expression features and phylogenetic relationships of NAC genes. NAC030/VND7 (At1g71930) and its ortholog Solyc06g065410.2 peaked within phase II, implying xylem formation. However, they were not identified as variant genes because of the relatively small variation amplitude. This may be due to the heterogeneous sampling location and/or narrow distribution of these types of genes, for which changes in expression levels were only slight. This was also the case for phloem-specific NAC020 and NAC086 genes. This also shows that although some genes were confirmed by experiments as good markers, they are not necessarily suitable as indicators of phenotypic changes based on expression levels. Evidence obtained from microstructure analysis should be included. Besides, NAC071 (At4g17980) and the orthologous gene Solyc08g077110.2 were expressed at their highest levels in GT_AT samples, and moderately high levels in GT_SL samples, and they also peaked during phase II. Recent studies reported that this Arabidopsis gene is a key regulator that promotes cambial cell formation (Matsuoka et al., 2021). This indicates that it may represent a switch point between the wounding response and cambium activation. Moreover, all the above-mentioned genes were expressed highly in grafted samples, and more so separated top samples than separated bottom samples, implying induction by auxin signaling, consistent with previous studies (Melnyk et al., 2018; Xie et al., 2019).

Therefore, we propose that two conserved biological processes play important roles. One is the transition from phase I to phase III, involving blocking of vascular meristem organization and upregulation of secondary metabolism, eventually forming cell wall structures. The other is phase II, involving cell division and vascular differentiation within the callus. Since the transition in the grafted bottom sections lagged behind that in the grafted top, and since gene expression levels in phase II were higher in the top sections than the bottom, both processes appear to be strongly affected by the hormone and metabolite status of tissue position above or below the graft junction. Very recently, some breakthrough advances in graft-related fields have been reported, including wound-induced cambium formation, the roles of auxin and sugars in activating vascular regeneration, and cell wall reconstruction facilitating cell-cell adhesion in a broad species range (Melnyk et al., 2018; Notaguchi et al., 2020; Matsuoka et al., 2021). Our results support these mechanisms at an evolutionary level. Importantly, comparative analysis of graft healing conservation may provide ways for applying these mechanisms to a wide range of species.

## Data Availability Statement

Raw data of RNA-seq transcriptomes of tomato and Arabidopsis can be obtained by the database of NCBI Sequence Read Archive (SRA) with the BioProject numbers PRJNA419306, PRJNA528328, and PRJNA645644.

## Author Contributions

LX and JG conceived and designed research. LX and QC analyzed the data and wrote the manuscript. JT, LP, YL, JL, FL, and SZ participate in material preparation and data analysis. All authors read and approved the manuscript.

## Conflict of Interest

The authors declare that the research was conducted in the absence of any commercial or financial relationships that could be construed as a potential conflict of interest.

## Publisher’s Note

All claims expressed in this article are solely those of the authors and do not necessarily represent those of their affiliated organizations, or those of the publisher, the editors and the reviewers. Any product that may be evaluated in this article, or claim that may be made by its manufacturer, is not guaranteed or endorsed by the publisher.

## Footnotes

1
http://www.arabidopsis.org/

2
http://www.phytozome.net/

3
http:/pfam.xfam.org/

## Supplementary Material

Supplementary Figure 1 **(A)** The top 5,000 variable genes (blue colored). **(B)** The soft threshold (power) selection for WGCNA.

Supplementary Figure 2 Log2FPKM values of genes in each module.

Supplementary Figure 3 ML tree and expression patterns of all NAC homologs in Arabidopsis and tomato.

Supplementary Figure 4 Log2FPKM values of genes in different species and different sample types. The significances of Kappa consistency tests between two species within each clade were marked with * (*p* < 0.05).

Supplementary Table 1 Gene expression levels (log2FPKM) of 11787 orthologous pairs in all samples.

Supplementary Table 2 197 NAC genes in Arabidopsis and tomato genomes and their expression levels (log2FPKM) in all samples.
