# Supplementary figures and images for: Conserved Regulatory Pathways for Stock-Scion Healing Revealed by Comparative Analysis of Arabidopsis and Tomato Grafting Transcriptomes
Source: Front Plant Sci. 2022 Feb 24;12:810465. doi: 10.3389/fpls.2021.810465 (PMC8908109; doi:10.3389/fpls.2021.810465)

A

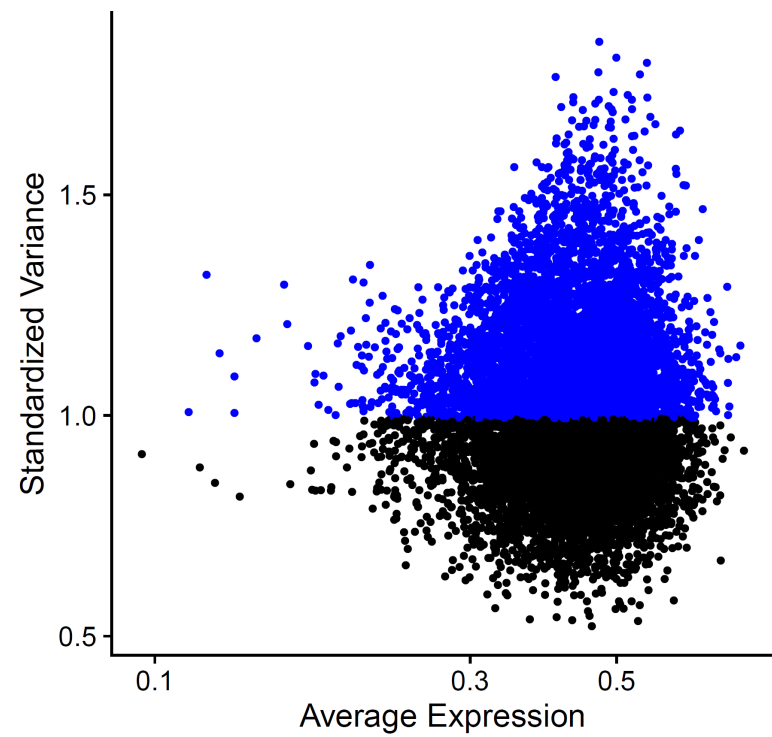

B

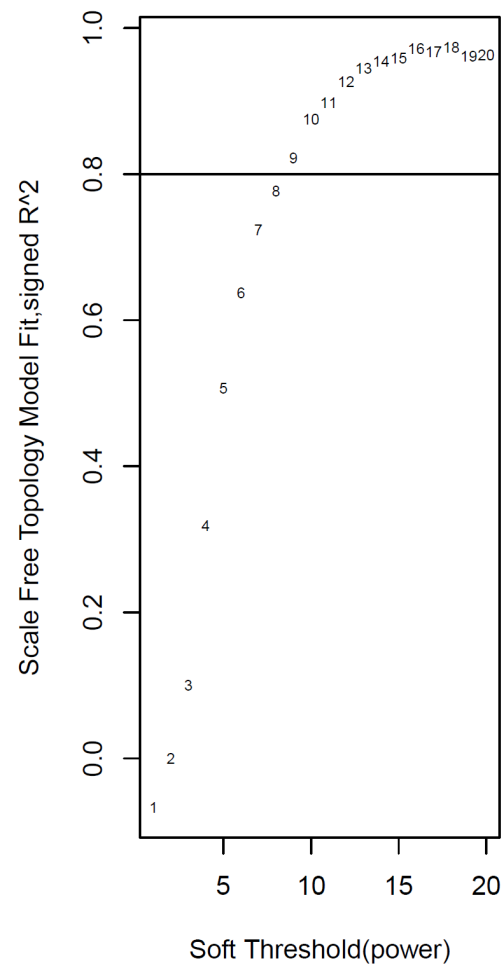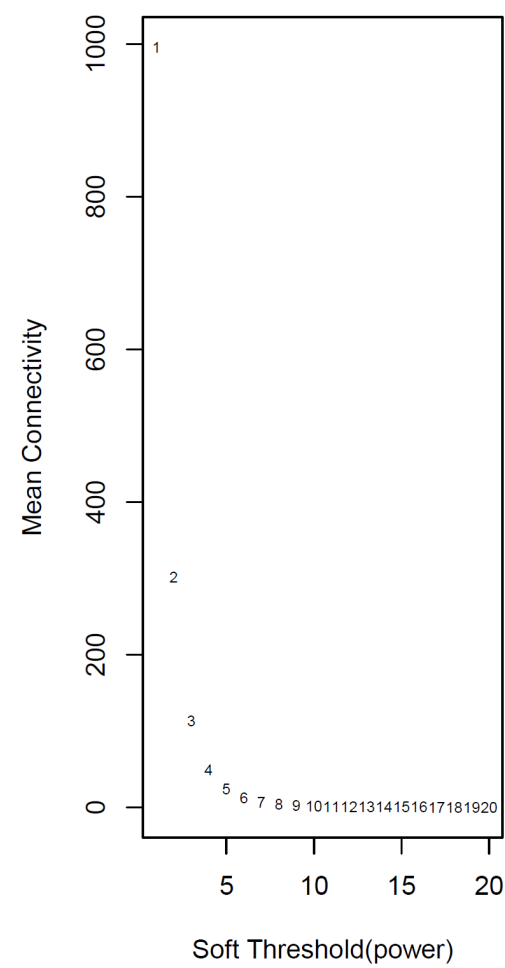

Supplement: Supplementary Figure 1 — (A) The top 5,000 variable genes (blue colored). (B) The soft threshold (power) selection for WGCNA. [file Image_1.pdf]

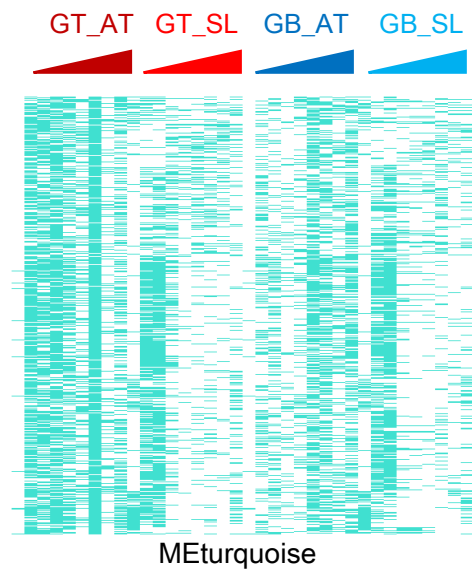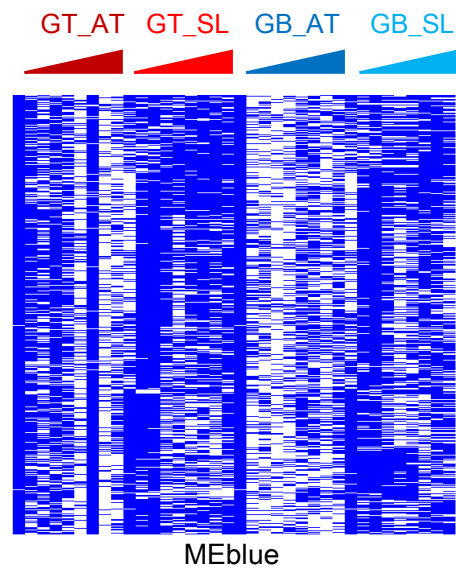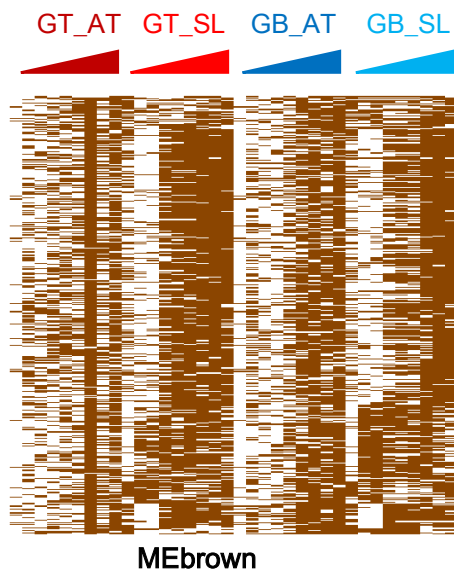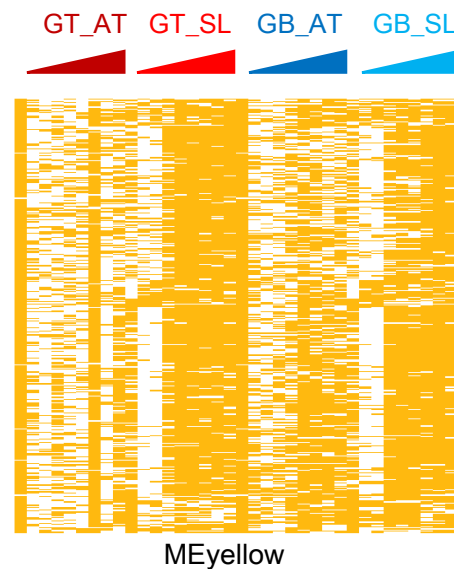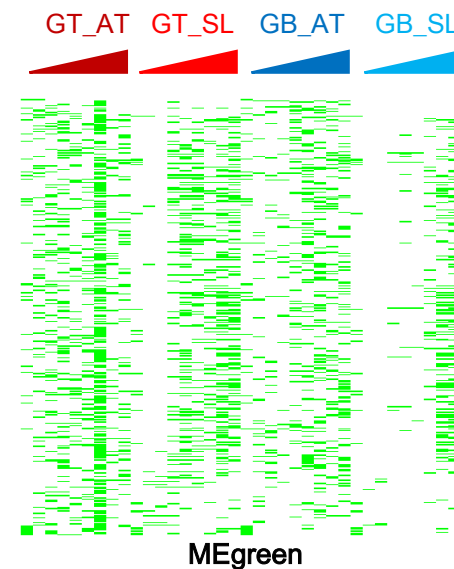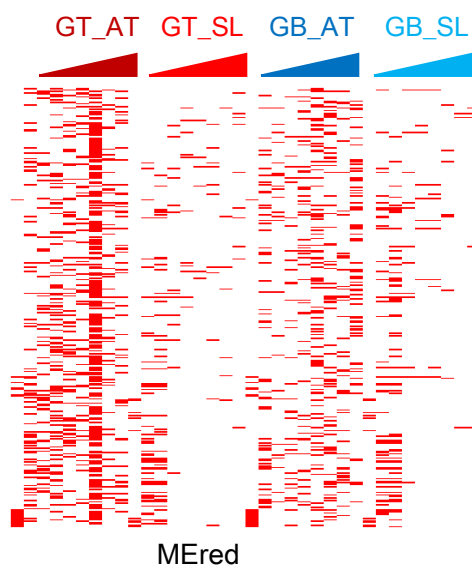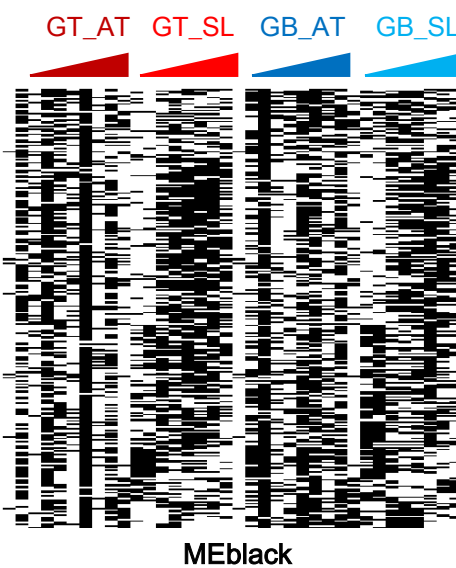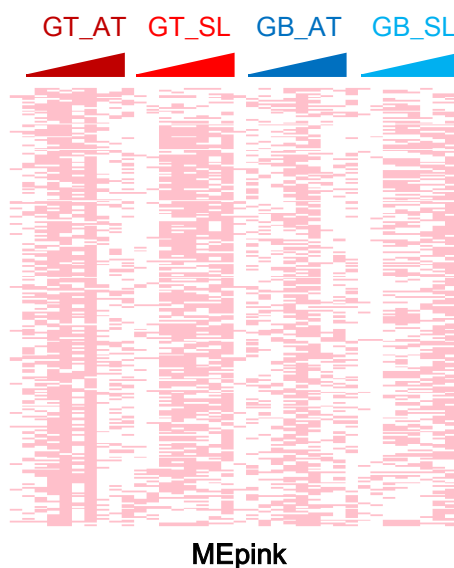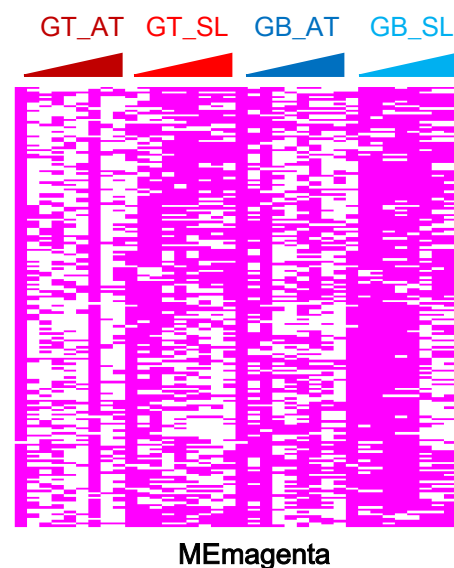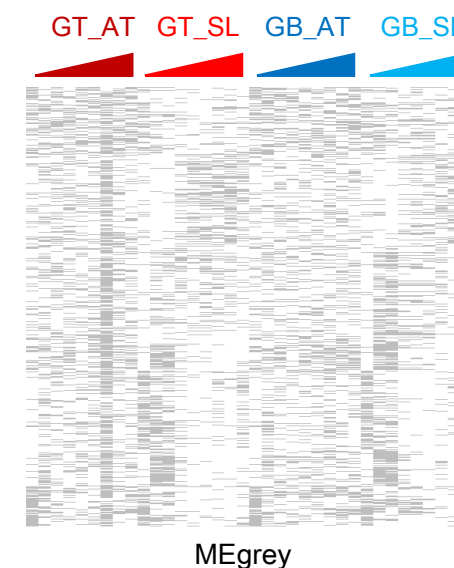

Supplement: Supplementary Figure 2 — Log2FPKM values of genes in each module. [file Image_2.pdf]

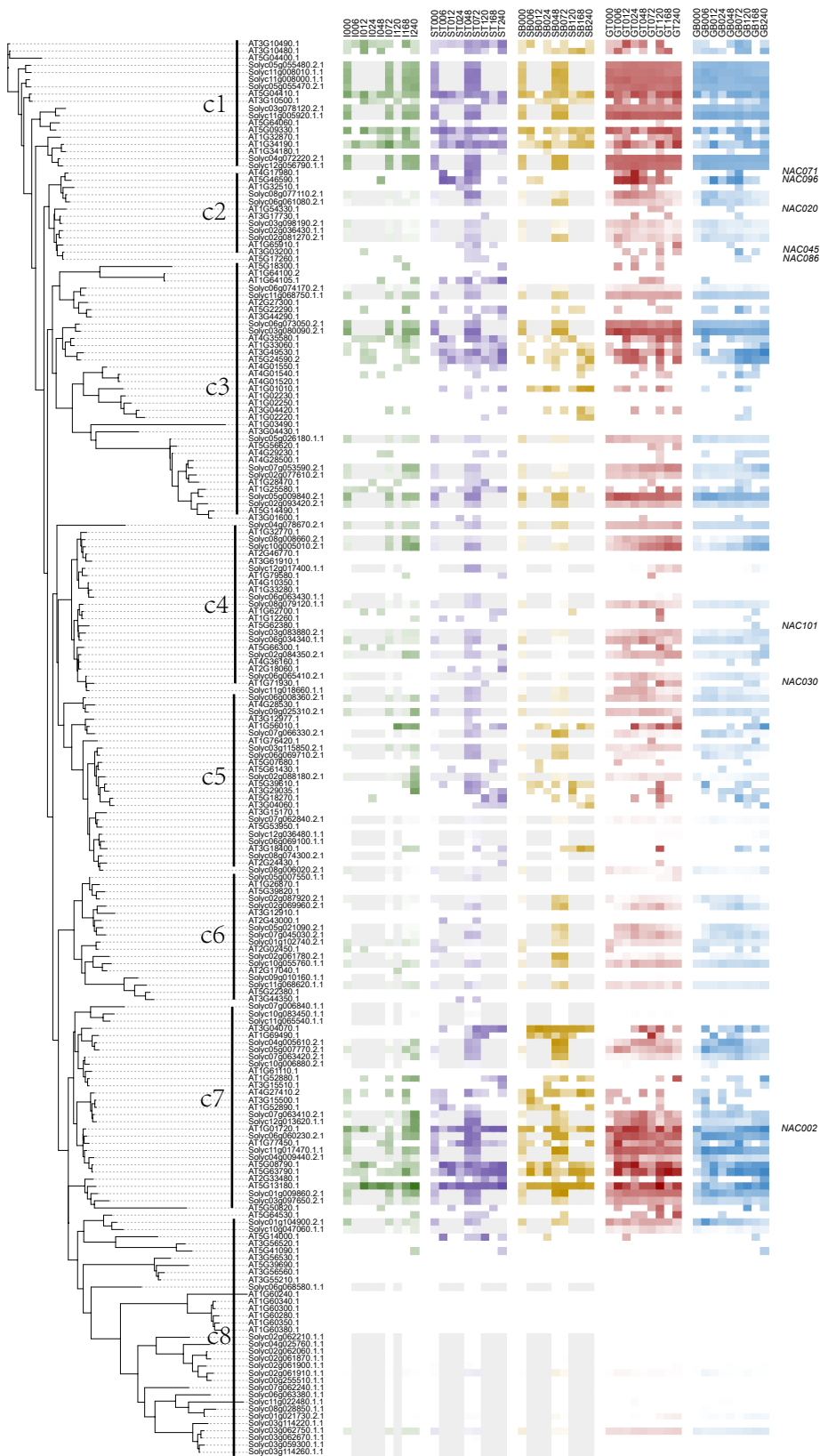

Supplement: Supplementary Figure 3 — ML tree and expression patterns of all NAC homologs in Arabidopsis and tomato. [file Image_3.pdf]

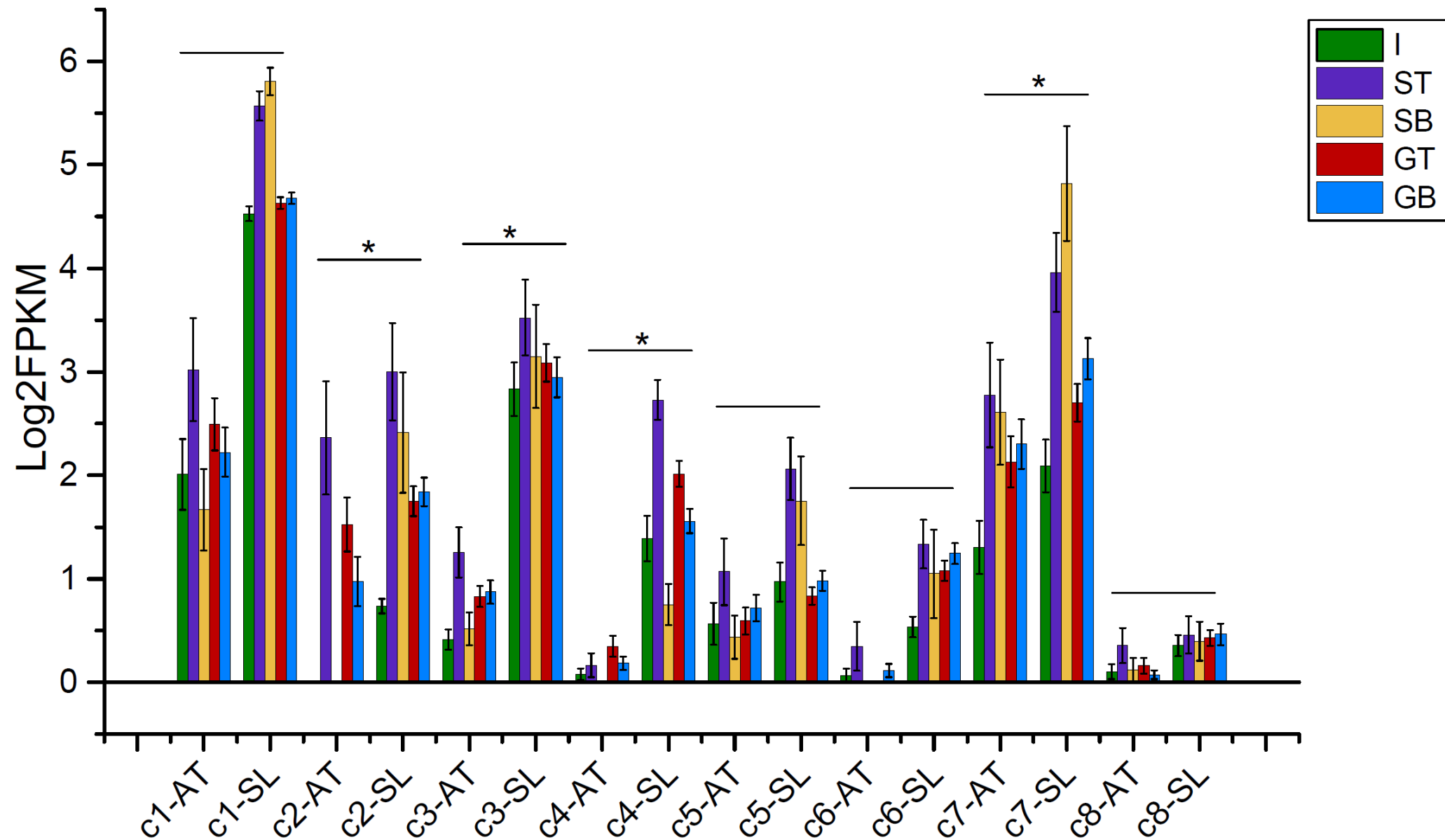

Supplement: Supplementary Figure 4 — Log2FPKM values of genes in different species and different sample types. The significances of Kappa consistency tests between two species within each clade were marked with * (p < 0.05). [file Image_4.pdf]
